# An overview of case reports and case series of pulmonary actinomycosis mimicking lung cancer: a scoping review

**DOI:** 10.3389/fmed.2024.1356390

**Published:** 2024-03-08

**Authors:** Amin Khoshbayan, Nour Amirmozafari, Shiva Mirkalantari

**Affiliations:** Department of Microbiology, School of Medicine, Iran University of Medical Sciences, Tehran, Iran

**Keywords:** actinomycosis, pulmonary actinomycosis, lung cancer, *Actinomyces* species, diagnosis

## Abstract

**Background:**

Pulmonary actinomycosis (PA) is a rare type of *Actinomyces* infection that can be challenging to diagnose since it often mimics lung cancer.

**Methods:**

Published case reports and case series of PA in patients with suspicion of lung cancer were considered, and data were extracted by a structured search through PubMed/Medline.

**Results:**

After analyzing Medline, 31 studies were reviewed, from which 48 cases were extracted. Europe had the highest prevalence of reported cases with 45.1%, followed by Asia (32.2%), America (19.3%), and Africa (3.2%). The average age of patients was 58.9 years, and 75% of all patients were above 50 years old. Male patients (70%) were predominantly affected by PA. The overall mortality rate was 6.25%. In only eight cases, the causative agent was reported, and *Actinomyces odontolyticus* was the most common isolated pathogen with three cases. Based on histopathological examination, 75% of the cases were diagnosed, and the lobectomy was performed in 10 cases, the most common surgical intervention. In 50% of the cases, the selective antibiotics were intravenous and oral penicillin, followed by amoxicillin (29.1%), amoxicillin-clavulanic acid, ampicillin, levofloxacin, and doxycycline.

**Conclusion:**

The non-specific symptoms resemble lung cancer, leading to confusion between PA and cancer in imaging scans. Radiological techniques are helpful but have limitations that can lead to unnecessary surgeries when confusing PA with lung cancer. Therefore, it is important to raise awareness about the signs and symptoms of PA and lung cancer to prevent undesirable complications and ensure appropriate treatment measures are taken.

## Introduction

*Actinomyces* species are Gram-positive bacteria with anaerobic and facultative microaerophilic metabolism that typically colonize the oropharynx, urogenital tract, or gastrointestinal system (1, 2). Actinomycosis is generally considered an endogenous infection. Although the bacteria are initially colonized on the surface of the mucosa, they can reach the deeper tissues through any disruption of the mucosal barrier caused by procedures such as trauma, surgical intervention, or foreign bodies (2–4). Actinomycosis is a rare and granulomatous disease that progresses slowly and creates sinus tract fistulae in a chronic form with a slow progression that creates sinus tract fistulae in a chronic form. It has been known for more than 150 years, and the most common causative agent is *Actinomyces israelii* (5, 6). In recent years, the frequency of all forms of actinomycosis has decreased, possibly as a result of the enhancement of oral hygiene and antibiotic therapy upon infection suspicion (1). However, there is no solid proof to support the effectiveness of such actions in reducing the incidence of colonization and mild periodontal infection with *Actinomyces* species (4, 6).

The common forms of actinomycosis are cervicofacial, abdominal, pelvic, and pulmonary. Moreover, on rare occasions, the spread of local infection through hematogenous dissemination may lead to the development of actinomycotic lesions in the lungs. Despite anatomic barriers, *Actinomyces* can spread and eventually invade the pleura, resulting in empyema formation.

With the improvement of oral hygiene and the availability of effective antibiotics, the severity of PA manifestation has become less severe. Furthermore, if diagnosis and treatment are not performed correctly, it can spread into the chest wall and create a pleuro-cutaneous fistula and destruction of vertebrae and ribs (1, 2, 7, 8). The diagnosis of pulmonary actinomycosis (PA) is quite challenging, and the delay in diagnosis can last for 6 months. PA usually results in the formation of nodules, consolidation, or mass that can often be mistaken for lung cancer. Therefore, PA could be misdiagnosed as lung cancer, lung abscess, or tuberculosis (9, 10). Due to non-specific laboratory and clinical features, it is usually challenging to differentiate PA from lung malignancy. Moreover, the most common initial diagnosis of PA among physicians is lung cancer (10–14). The common signs and symptoms of PA are fever, chest pain, hemoptysis, shortness of breath, and a productive cough (15, 16). Furthermore, the severity of PA manifestations has become less severe with the enhancement of oral hygiene and the availability of effective antibiotics.

Dealing with PA can be challenging due to its difficult diagnosis. However, if more people are aware of this infection, it could lead to an easier diagnosis and prevent undesired complications such as unnecessary surgeries and treatment with the wrong medication. To study this, we conducted a scoping review that explored the clinical, epidemiological, diagnostic, and therapeutic features of PA cases that were initially suspected of lung cancer.

## Methods

### Search strategy

In the current study, a Medline search (via PubMed) was performed on 4 December 2022. The keywords were chosen from the National Library of Medicine’s Medical Subject Heading (MeSH) terms, titles, and abstracts through Boolean operators (and/or) including “Pulmonary Neoplasms” or “Lung Neoplasm” or “Lung Cancer” or “Pulmonary Cancer” or “Cancer of Lung,” and (Actinomyc*). The present study was conducted according to the PRISMA extension for scoping reviews.

### Inclusion and exclusion criteria

All case reports and case series studies were included where the cancer was initially suspected in the diagnosis process, and irrelevant articles (review articles, conference abstracts, and studies with unclear results and insufficient data) were excluded.

### Study selection and data extraction

The titles, abstracts, and full texts of all included studies were reviewed independently by two authors (AKH and SHM). The search was limited to English-published studies, and any disagreements among authors were resolved through discussion and consensus. The data extracted from each study included the first author’s name, publication year, country, sex, age, *Actinomyces* species, treatment, surgery or puncture drainage for biopsy, diagnosis method, radiologic finding, patient outcome, and additional findings.

## Results

### Epidemiology

**Figure 1:**
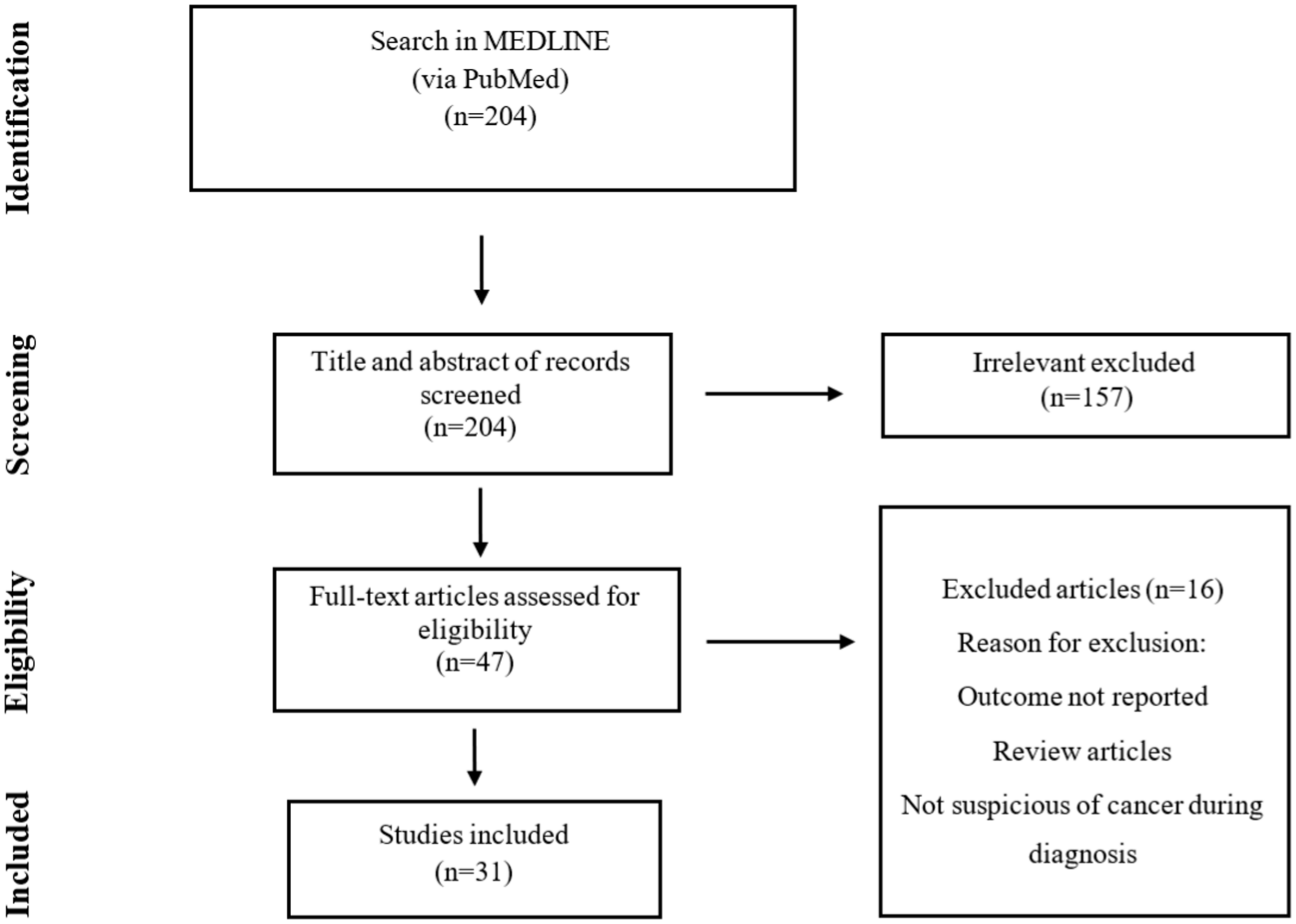
Flowchart of publication selection and their inclusion in the scoping review.

**Figure 2:**
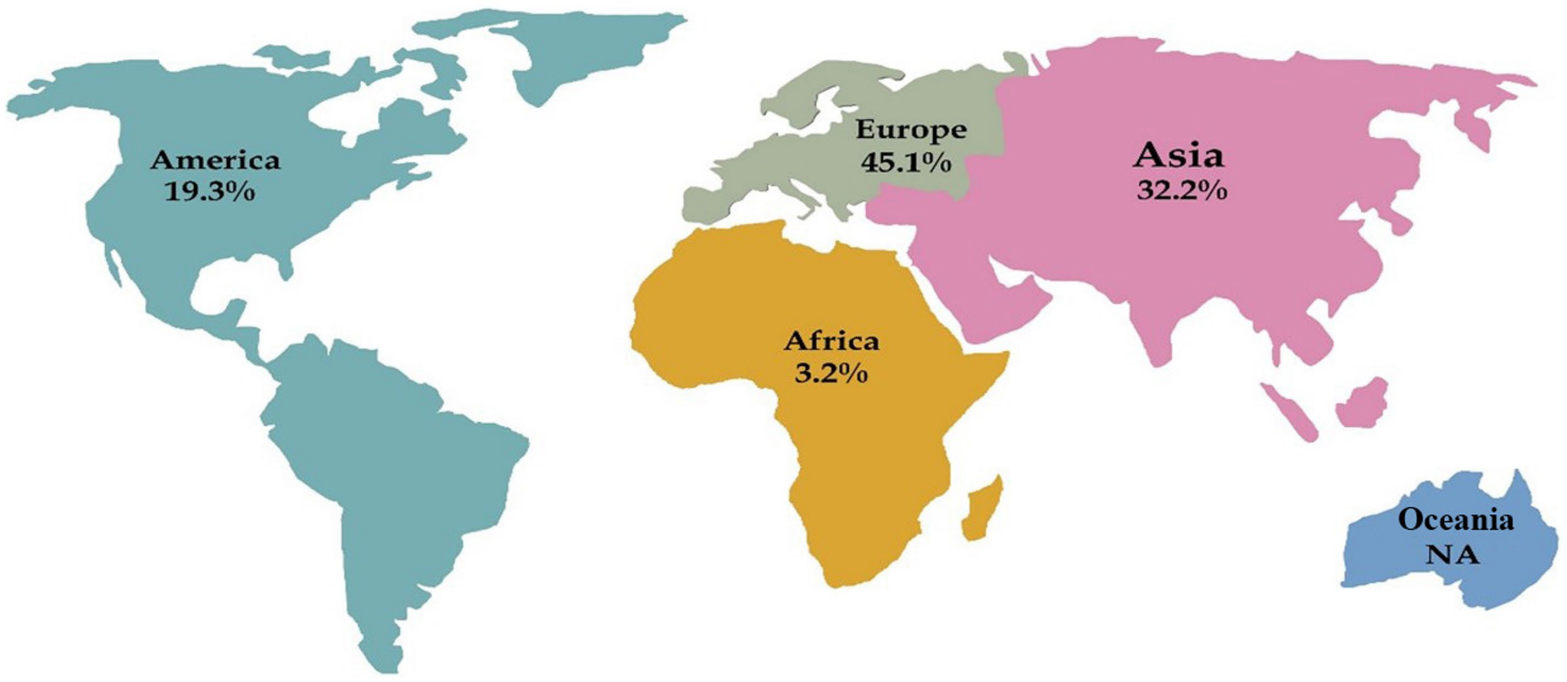
Distribution of pulmonary actinomycosis cases in each continent. NA: not available.

Our search of the Medline database yielded a total of 204 hits, we reached 31 studies, of which 48 cases were included in the final analysis (Figure 1). These cases were reported from Poland, Malaysia, India, Korea, Germany, Italy, and the Netherlands (one study), China, Spain, and Turkey (two studies), Japan and Greece (four studies), and the USA (six studies). Furthermore, there were four case series from Japan, Italy, Germany, and Tunisia (one study). Accordingly, Europe had the highest share of reported studies with 45.1% (14 studies), followed by Asia with 32.2% (10 studies), America with 19.3% (6 studies), and Africa with 3.2% (one study). No cases were identified from Oceania (Figure 2).

### Outcome and etiology

Overall, PA showed low mortality, and only three patients died. A 59-year-old female developed lung adenocarcinoma following an initial PA diagnosis. The patient died eventually after chemotherapy failure. In another 50-year-old male patient, recovery happened, although, after 1.5 years, the patient died from a massive gastrointestinal hemorrhage. The third patient was an 83-year-old male who died despite antibiotic treatment with penicillin.

Our results showed that only 25 and 70% of the patients were female and male, respectively, and in two cases, sex was not reported. The mean age of patients was 58.9, within the range of 36–86, and 75% of all patients were above 50 years old (Tables 1, 2).

**Table 1:** Epidemiological, clinical, diagnosis, and therapeutic features of patients with pulmonary actinomycosis from individual case reports.

Author and references	Country, year of publication	Sex/age	Species	Treatment	Surgery or puncture drainage for biopsy	Diagnosis method	Radiologic finding	Patient outcome	Additional findings
Qiu et al. (17)	China, 2015	41/M	*A. odontolyticus*	IV Piperacillin-sulbactam and levofloxacin followed by oral cefuroxime and levofloxacin	CT-guided lung puncture biopsy	Culture	PET/CT demonstrated a mass in the upper lobe of the right lung	Recovered	-
Aydin et al. (18)	Turkey, 2022	54/F	NR	NR	Lobectomy	HE	PET/CT scan shows a lesion with irregular borders in the anterior segment of the left lung upper lobe	NR	-
Miyazaki et al. (19)	Japan, 2022	64/M	NR	Amoxicillin	Surgical extirpation of the mass	HE	CT scan showed mass-like consolidation in the left upper lobe	Recovered	-
Asif et al. (20)	USA, 2021	75/F	*A. odontolyticus*	Liposomal amphotericin and oral amoxicillin	CT-guided biopsy	Culture	chest CT revealed dense consolidation in the right lower pulmonary lobe (RLL) with some mild hilar and mediastinal adenopathy	Recovered	Co-infection with *Coccidioides posadasii/immitis*
Drozdowicz1 et al. (21)	USA, 2021	59/F	*Actinomyces* and *Prevotella* spp	Amoxicillin/Clavulanic acid	BAL and bronchial brushing	NR	CT was suggestive of malignancy, with a mass in the left upper lobe obstructing the lingular bronchus pleural effusion	Initial recovery but after chemotherapy failure patient died	After the initial actinomycosis diagnosed, pathology confirmed lung adenocarcinoma
Tanaka et al. (22)	Japan, 2020	60/M	*A. israelii*	Piperacillin /tazobactam followed by oral penicillin	CT-guided biopsy, and bronchoscopy	Culture	Chest CT showed a 4.0 cm mass-like lesion in the lingular segment of the left lung.	Recovered	-
Tanino et al. (23)	Japan, 2020	86/M	NR	Tosufloxacin and clarithromycin	Thoracoscopic lung biopsy	HE	CT revealed a 48 × 42-mm tumor shadow in the right middle lobe	Recovered	PNLH was present adjacent to *Actinomyces*
Karadeniz et al. (24)	Turkey, 2019	49/M	NR	Amoxicillin	Left lower lobectomy	Gram and PAS staining	PET shows parenchymal lesions with a size of 4.2 × 2.2 cm accompanied by air bronchograms in the left lower lobe mediobasal segment, band-like atelectasis in the distal region, and subcarinal lymph nodes	Recovered	-
Balis et al. (25)	Greece, 2019	62/M	*A. odontolyticus*	INH – RIF – ETB – PZA for 8 weeks following INH – RIF for 18 weeks and doxycycline 200 mg per day for 12 months.	Cryoadhesion	HE and 16S rRNA gene sequencing	CT scan shows a well-defined mass in the middle lobe extending to the right lower lobe with surrounding airspace disease	Recovered	Co-infection with *M. tuberculosis* (BAL culture)
Oikonomidis et al. (26)	Greece, 2019	68/M	NR	Penicillin G and followed by doxycycline	Bronchoscopic biopsy	PAS	CT thorax was performed, revealing patchy air space consolidation in the posterior segment of the left lower lobe	Recovered	-
Blázquez et al. (27)	Spain, 2019	65/M	NR	NR	Endoscopic biopsy by cryoprobe	HE	CT showed thickening of the distal wall of the left main bronchus which had acquired a nodular morphology	NR	-
Ding et al. (28)	China, 2018	70/M	NR	Levofloxacin and mezlocillin/sulbactam	Bronchoscopy	HE	CT revealed left-sided pleural effusion and a mass in the lower left lung	Recovered	-
Habib et al. (29)	USA, 2018	74/M	*A. viscosus*	Amoxicillin at first and continued by penicillin and methotrexate	Neck abscess drainage	Culture	CT showed the presence of left upper lobe lung mass along with multiple pulmonary nodules and multiple low-density circular structures scattered MRI of the brain revealed scattered rim-enhancing lesions with surrounding edema throughout the brain in the left frontal lobe	Recovered	Patient treated with methotrexate due to psoriatic arthritis brain lesions were secondary to a disseminated infection
Grzywa-Celińska et al. (30)	Poland, 2018	77/NR	NR	Penicillin G and amoxicillin	Peribronchial lung biopsy	HE	CT scan shows spicular consolidation at the base of segment 2 of the right lung and an area of parenchymal consolidation in segment 8 of the right lung	Recovered	-
Papakonstantinou et al. (31)	Greece, 2018	76/NR	NR	NR	Right lower lobectomy	HE	PET-CT revealed a right lower lobe lesion measuring 5.6 cm in diameter with high metabolic activity	Recovered	-
Boo et al. (32)	Malaysia, 2017	49/M	NR	IV penicillin followed by oral penicillin	CT-guided biopsy	HE	CT showed a mass over the left lower zone with a satellite lesion over the left upper zone, and left basal loculated effusion	Recovered	-
Laguna et al. (33)	Spain, 2016	76/F	NR	NR	Right lower lobectomy	HE	PET/CT, which showed the lesion in the RLL with a maximum standardized uptake value (SUV) of 4	NR	Superinfection of *Actinomyces* associated with a foreign body (fish bone)
Bunkar et al. (34)	India, 2016	50/F	NR	IV penicillin followed by oral amoxicillin / clavulanic acid	An ultrasound-guided Trucut biopsy	PAS	Contrast-enhanced chest tomography (CECT) showed a heterogeneously enhancing mass lesion, involving the apicoposterior segment	Recovered	-
Imanishi et al. (35)	Japan, 2016	43/M	NR	Ampicillin followed by oral amoxicillin	Bronchoscopy	HE	CT showed an irregular-shaped mass in the left inferior lobe, with airway stenosis of the lobar bronchus	Recovered	-
Katsenos et al. (36)	Greece, 2015	67/M	NR	Penicillin followed by amoxicillin	EBUS-guided transbronchial biopsies	Gram staining	Chest radiograph showed an infiltrate in the right upper lobe with associated mild pleural thickening	Recovered	-
Katsenos et al. (36)	Greece, 2015	70/F	NR	Penicillin followed by amoxicillin	Rigid forceps by bronchoscopy	HE	CT scan shows a right hilar mass compressing the bronchus intermedius with accompanying dense airspace opacification of the right lower lobe and atelectasis	Recovered	-
Park et al. (37)	Korea, 2014	46/M	*A. meyeri*	IV penicillin G and metronidazole followed by oral amoxicillin	Stereotactic biopsy of brain abscesses	Gram staining, culture, and 16SrRNA sequencing	CT confirmed a mass in the left lobe with a speculated border and peripheral subsegmental atelectasis. Magnetic resonance brain scan showed a 3.5 cm necrotic mass with peripheral rim enhancement in the left frontoparietal lobe, and enhancing nodular lesion in the subcortical white matter of the left parietal lobe	Recovered	Pulmonary actinomycosis with brain abscess. *Actinomyces* spp., *Propionibacterium acnes*, and *Fusobacterium nucleatum* were grown. *A. meyeri* confirmed by16S rRNA sequencing
Fichte et al. (38)	Germany, 2013	55/M	NR	Ampicillin/sulbactam	Surgery with approach to the cervicothoracic junction	HE	CT of the cervical spine showed a destructing process in the vertebrae C7 (partial) and T1 MRI localizers showed an apical lung mass on the right side	Recovered	Vertebral and Pulmonary Actinomycosis growth of *Actinobacillus actinomycetemcomitans* was observed.
Godfrey et al. (39)	USA, 2012	62/F	NR	Oral Penicillin VK	Bronchoscopy	HE	CT scan shows a nodule in the right lower lobe and a stable right suprahilar soft tissue opacity consistent with postradiation change	Recovered	The patient had a history of stage III B lung squamous cell carcinoma and chemotherapy thirty-three months before infection
Elkambergy et al. (40)	USA, 2009	54/M	*Actinomyces naeslundii*	Oral penicillin V	Thoracotomy	Culture	CT scans of the chest revealed an irregular density in the apical portion of his right upper lobe	Recovered	Patient had a history of stage III rectal adenocarcinoma and chemotherapy and radiation were completed 6 months prior to infection.
Andreani et al. (41)	Italy, 2012	62/M	NR	Amoxicillin	Transbronchial needle aspiration	Grocott methenamine silver stain	Mass in the surgical bronchial stump	Recovered	Patient had a history of throat cancer
Colmegna et al. (42)	USA, 2003	50/M	*A meyeri*	Intravenous penicillin followed by oral amoxicillin	CT-guided fine-needle aspirate	16S rDNA sequencing	The mass was pleura-based and associated with hilar lymphadenopathyMagnetic resonance brain scan showed multiple thin-walled, ring-enhancing lesions in both cerebral hemispheres	Recovered, but after approximately one and half year patient died from a massive gastrointestinal hemorrhage	*Actinomyces* spp., *Peptostreptococcus* spp., and *Fusobacterium* spp. By 16S rDNA sequencing confirmed
Neijens et al. (43)	Netherlands, 1996	42/M	NR	Penicillin G followed by oral feneticilline	Left thoracotomy	HE	CT scan shows left paravertebral mass with extension to the contralateral pleural space, erosion of the vertebral body and intrapulmonary mass	Recovered	-

**Table 2:** Epidemiological, clinical, diagnosis, and therapeutic features of patients with pulmonary actinomycosis from individual case series.

Author and references	Country, year of publication	Sex/age	Species	Treatment	Surgery or puncture drainage for biopsy	Diagnosis method	Radiologic finding	patient outcome	Additional findings
Boudaya et al. (44)	Tunisia, 2012	36/M	NR	IV penicillin G followed by oxacillin	Biopsy by mediastinotomy	HE	Expansive process parietal and mediastinal involvement and nodes	Recovered	-
Boudaya et al. (44)	Tunisia, 2012	46/M	NR	IV ampicillin followed by amoxicillin	Left upper lobe wedge resection	HE	Two suspected masses	Recovered	-
Boudaya et al. (44)	Tunisia, 2012	52/M	NR	Penicillin G followed by amoxicillin	Right lower lobectomy extended to diaphragm	HE	Suspected mass and mediastinal nodes	Recovered	-
Boudaya et al. (44)	Tunisia, 2012	44/F	NR	IV penicillin G followed by amoxicillin	Left lower lobectomy	HE	Cystic formation	Recovered	-
Schweigert et al. (45)	Germany, 2012	46/M	NR	Penicillin	Thoracotomy	HE	NR	Recovered	Patient had a history of urinary bladder cancer
Schweigert et al. (45)	Germany, 2012	58/M	NR	Penicillin	Right-sided thoracotomy	HE	NR	Recovered	Patient had a history of ischemic stroke 1 year previously and suffering from chronic obstructive pulmonary disease
Schweigert et al. (45)	Germany, 2012	40/M	NR	Penicillin	Middle lobe was resection	HE	CT showed advanced pleural empyema with pleural thickening and extended effusion as well as patchy parenchymal consolidations	Recovered	-
Andreani et al. (46)	Italy, 2009	68/M	NR	Amoxicillin / Clavulanic acid	Bronchial biopsy	HE	Opacity in right lobe, mediastinal adenopathy, “bronchial tree-in-bud” pattern	Recovered	Vegetables detected as foreign material
Andreani et al. (46)	Italy, 2009	54/F	NR	NR	Atypical resection	HE	Multiple opacities mimicking pulmonary metastases	Recovered	Patient had a history of breast neoplasm (14 years before) and kidney neoplasm metastases (5 years before)
Andreani et al. (46)	Italy, 2009	54/F	NR	NR	Lobectomy	HE	Mass-like consolidation mimicking a neoplasm	Recovered	-
Andreani et al. (46)	Italy, 2009	54/M	NR	NR	Lobectomy	HE	Mass-like consolidation mimicking a neoplasm in the left lower lobe	Recovered	Splenectomy and left atypical lung resection due to injury in motor vehicle crash
Andreani et al. (46)	Italy, 2009	83/F	NR	Amoxicillin / Clavulanic acid	Transthoracic biopsy	HE	Mass-like consolidation	Recovered	-
Endo et al. (47)	Japan, 2002	61/M	NR	Penicillin	Partial resection	HE	CT show Low attenuation area, spiculation and pleural thickening	Recovered	*-*
Endo et al. (47)	Japan, 2002	83/M	NR	Penicillin	Right lower lobectomy	HE	CT scan shows pleural indentation, low attenuation area, spiculation and pleural thickening	Died	*-*
Endo et al. (47)	Japan, 2002	52/M	NR	Erythromycin	Partial resection	HE	CT scan shows low attenuation area, spiculation and pleural thickening	Recovered	*-*
Endo et al. (47)	Japan, 2002	44/M	NR	Penicillin	Thoracoscopic partial resection	HE	CT scan shows low attenuation area, pleural indentation, transgression of interlobar fissure and pleural thickening	Recovered	-
Endo et al. (47)	Japan, 2002	73/F	NR	Penicillin	Lingular segmentectomy	HE	CT scan shows low attenuation area, spiculation and pleural thickening	Recovered	-
Endo et al. (47)	Japan, 2002	50/M	NR	Penicillin	S2 segmentectomy	HE	CT show spiculation and pleural thickening	Recovered	-
Endo et al. (47)	Japan, 2002	77/M	NR	NR	Thoracoscopic partial resection	HE	CT show spiculation and pleural indentation	Recovered	-
Endo et al. (47)	Japan, 2002	50/M	NR	NR	Left upper lobectomy	HE	CT scan shows low attenuation area, spiculation and pleural thickening	Recovered	-

M, male; F, female; HE, histopathological examination; NR, not reported; PNLH, pulmonary nodular lymphoid hyperplasia; PAS, periodic acid-Schiff; BAL, bronchoalveolar lavage; MRI, magnetic resonance imaging; NH, isoniazid; RIF, rifampin; ETB, ethambutol; PZA, pyrazinamidel; EBUS, endobronchial ultrasound.

Several patients in the study had various medical conditions in addition to PA. In one patient, pulmonary nodular lymphoid hyperplasia (PNLH) was diagnosed adjacent to the *Actinomyces* lesion, and lung adenocarcinoma was detected in one patient after PA diagnosis. Moreover, one of the patients had a history of treatment for lung squamous cell carcinoma, 33 months before the PA diagnosis. Another case had a history of treatment for rectal adenocarcinoma 6 months before the infection. Moreover, there were two patients with a history of throat and urinary bladder cancer, as well as one patient with a history of breast neoplasm (14 years before the infection) and kidney neoplasm metastases (5 years before the infection). Another patient had a history of treatment with methotrexate as well as a brain lesion following the disseminated infection. One patient had PA with a brain abscess. One patient tested positive for tuberculosis by culture of bronchoalveolar lavage fluid. Furthermore, another patient was involved in vertebral and PA with the growth of *Actinobacillus actinomycetemcomitans*. Additionally, in one patient, co-infection with *Coccidioides posadasii/immitis* was reported.

**Figure 3:**
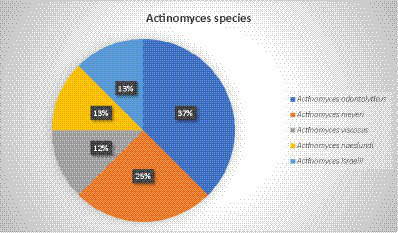
*Actinomyces* species related to pulmonary actinomycosis.

In addition to *Actinomyces*, *Prevotella* spp., *Propionibacterium acnes*, *Fusobacterium nucleatum*, *Peptostreptococcus* spp., and *Fusobacterium* spp. were also found in some patients. One patient had an ischemic stroke 1 year before the infection and suffered from chronic obstructive pulmonary disease. Another patient who was involved in a motor vehicle crash underwent splenectomy and atypical lung resection. The *Actinomyces* infection seemed to be related to foreign bodies, such as fish bones and vegetables, which were found in two patients. Most of the patients (79%) with PA were immunocompetent. Accordingly, in 83% of the cases, *Actinomyces* at the species level were not detected, and species identification was reported in only 8 cases. The most reported species was *Actinomyces odontolyticus*, with three cases, followed by *Actinomyces meyeri*, with two cases. *Actinomyces viscosus*, *Actinomyces naeslundii*, and *Actinomyces israelii* were found in only one case each. Culture was the most common detection method, while 16SrRNA sequencing was performed in two cases and 16SrDNA sequencing was used in one case (Figure 3).

### Diagnosis method

**Figure 4:**
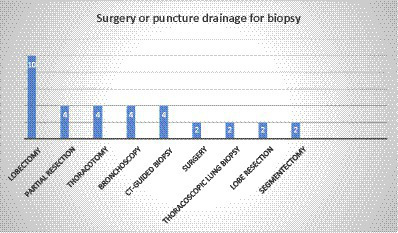
Distribution of individual methods for biopsy.

In most of the cases (75%, 36 out of 48), the diagnosis was based on the histopathologic examination (HE) of different types of specimens. The most common specimen was a lobectomy (10 cases), while partial resection, thoracotomy, bronchoscopy, and CT-guided biopsy were reported in 4 cases each. Moreover, surgery, thoracoscopic lung biopsy, lobe resection, and segmentectomy were observed in two cases (Figure 4). In two patients with brain involvement, a stereotactic biopsy of brain abscesses and neck abscess drainage was performed.

**Figure 5:**
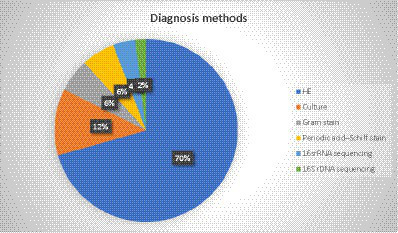
Different methods used for the diagnosis of pulmonary actinomycosis.

The bacterial culture was diagnostic only in 12.5% of the patients (six cases), as *Actinomyces* species are difficult to grow. Gram staining and periodic acid–Schiff stain were also reported in three cases, 16SrRNA sequencing in two cases, and 16SrDNA sequencing was reported in one case. Additionally, the diagnosis method was not reported in one case. Figure 5 represents the different methods used to diagnose PA.

### Treatment

**Figure 6:**
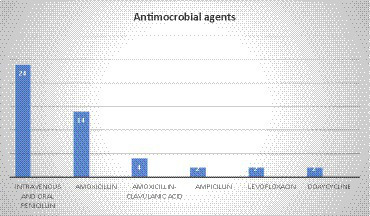
Antimicrobial agent used for the treatment of pulmonary actinomycosis.

In a total of 48 cases, the antimicrobial treatment was not mentioned in 9 cases. In the majority of cases (50%, 24 cases), treatment was administered via both intravenous and oral penicillin. Amoxicillin was the second most common agent with 29.1% (14 cases, 29.1%), followed by amoxicillin–clavulanic acid (4 cases), and ampicillin, levofloxacin, and doxycycline, each with 2 cases. Additionally, feneticilline, erythromycin, oxacillin, ampicillin/sulbactam, piperacillin–sulbactam, mezlocillin /sulbactam, metronidazole, tosufloxacin, clarithromycin, cefuroxime, and piperacillin /tazobactam were each reported in one case (Figure 6).

In another case, a patient had a co-infection with *Coccidioides posadasii/immitis*. The treatment plan consisted of liposomal amphotericin and oral amoxicillin, which proved to be successful in leading to the patient’s recovery. Another patient had a co-infection with *Mycobacterium tuberculosis,* and treatment was started with rifampin, ethambutol, pyrazinamide, and isoniazid for 8 weeks. It was followed by rifampin and isoniazid for 18 weeks and doxycycline for 12 months.

## Discussion

PA is a rare actinomycosis disease with a slow-progressing form of pulmonary infection, with a prevalence of 15% in all actinomycosis cases. This infection is often associated with the aspiration of oropharyngeal or gastrointestinal secretions (31, 48). It can involve both sexes and any age, but our findings showed that most of the infected patients were men and that 75% of patients were over 50 years of age. This is in correlation with other studies declaring that PA was more common in male patients and that the peak incidence of infection reached in the fourth to fifth decades of age (12, 24, 48). The high incidence of PA in male patients could be partly related to poorer oral hygiene and the occurrence of more facial trauma (9, 48).

The findings of a chest computed tomography scan (CT scan) of actinomycosis are non-specific and resemble necrotic lung malignancy. This condition is characterized by chronic segmental airspace consolidation with low-attenuation areas that have peripheral enhancement (7, 10, 49). Additionally, cavitations, shadowing, and pleural effusion with cavitary lesions are also typical features of PA that can be misdiagnosed as tuberculosis (28). Similarly, our results revealed that in most of the cases, CT scans were useful for diagnosis, but not always conclusive. The scan showed air space consolidation, mass in the middle lobes that could be misinterpreted as malignancy, pleural empyema, and opacity in the lobes, which could mimic pulmonary metastases. Moreover, positron emission tomography-computed tomography (PET-CT) is a helpful imaging technique to differentiate benign lesions from malignant ones, but there is limited information on PET-CT findings about PA (10, 50).

This technique was used in five cases and showed lesions and masses in lung lobes. Nevertheless, there is minimal information about PET-CT findings on PA. This diagnostic method has also encountered some issues. According to Choi et al., PET-CT is not an ideal tool for the differentiation of PA from lung cancer because of its high fluorodeoxyglucose (FDG) uptake. Consequently, physicians may mistake the high FDG uptake in favor of lung malignancy over PA. Therefore, clinicians must carefully evaluate the need for lung resection surgery when PA is suspected (17, 41, 51).

Some pulmonary infections, such as tuberculosis, aspergilloma, and histoplasmosis, can create false positive results due to their high metabolic uptake. Furthermore, FDG uptake has been observed in actinomycosis, leading to a mimicry of pulmonary malignancies (10, 24, 45, 52).

Although the diagnosis of PA could be delayed, our results showed that the mortality rate was only 6.25% and the overall outcome was acceptable. Similarly, a recent study in China showed that 75.9% of patients fully recovered, while another study in Korea reported a 98% recovery rate among 94 patients with PA (12). Therefore, PA seemed to have a good prognosis with a low rate of mortality because of antibiotic treatment and surgical intervention.

Actinomycosis might coexist with lung cancer, making the diagnosis even harder (9). Among the patients we studied, six had a history of cancer, and two had lung cancer. Although actinomycosis is unusual in immunosuppressed patients, immune system abnormalities may be a facilitating factor for the development of infection. However, the exact relationship between the two conditions is not yet fully understood (53). Interestingly, we found three cases in our research where PA involvement was diagnosed after cancer treatment (54–56). In one of the cases, the patient has been treated with bevacizumab for advanced non-small-cell lung cancer. After 36 months of bevacizumab maintenance, the patient was diagnosed with actinomycosis in the right lung. Bevacizumab was discontinued, and the patient was treated with amoxicillin–clavulanic acid. Unfortunately, the patient passed away after 3 months (54). The diagnostic method was culture in only 12% of the cases. Currently, positive culture in PA is rare due to the challenges of culturing anaerobic bacteria. Previous antibiotic treatments and bacterial overgrowth can also complicate matters. In addition, the evidence suggests that using normal saline, which is usually used for bronchoalveolar lavage, can prevent *Actinomyces* growth (9, 45, 57, 58). On the other hand, isolating *Actinomyces* may be crucial to distinguishing nocardiosis or botryomycosis from actinomycosis, which is usually difficult to differentiate morphologically. As a result, the direct culture of biopsy material in both aerobic and anaerobic blood culture media can improve culture sensitivity (59). The accurate diagnosis of PA depends on HE, as radiologic imaging and culture may not be conclusive. Without histological or microbiological confirmation, misdiagnosis can be fairly common (48, 60). However, sulfur granules in biopsy can be essential and suggestive, but not specific. On the other hand, when a small amount of tissue is biopsied, sulfur granules can be missed (60, 61). Nevertheless, granulomas and multinucleated giant cells can be observed in some cases. These morphological shapes are not specific, and other pathogens such as *Nocardia* spp. and some fungal and parasitic infections can cause similar observations. Furthermore, Grocott methenamine silver staining can identify the branching microorganism that is specific for the existence of actinomycosis infection (6, 62, 63).

Furthermore, surgical intervention may be necessary for diagnosis and treatment if lung cancer cannot be ruled out (31, 47). Endo et al. declared that a conclusive differential diagnosis between PA and necrotic lung cancer might be possible only when the surgical restriction specimen is sent for HE (47).

Furthermore, it has been shown that surgery can be avoided in most cases of thoracic actinomycosis, and long-term intravenous penicillin therapy leads to a good prognosis. However, early surgical intervention may lead to equally good or better outcomes by shortening the antibiotic therapy period (13, 64).

Our results showed that in 75% of the cases diagnosed with HE, and similarly, in 94 cases in Korea, all PA patients were diagnosed with HE (12). Altogether, HE is an essential method for the correct diagnosis of PA (17). Moreover, the new approach of using molecular methods in diagnosis can be helpful in the detection of PA, as in one case, HE and 16SrRNA sequencing were used together for diagnosis. This was a complicated case, and the patient had a co-infection with PA and tuberculosis. The molecular method also led to the identification of a bacterial species, which was *A. odontolyticus*.

Moreover, 16SrRNA sequencing helped the diagnosis of *A. meyeri* in a complicated case of PA involvement with a brain abscess. In another case, the diagnosis made by 16SrDNA demonstrated that *A. meyeri* was a causative agent of PA. Furthermore, 16SrRNA is a component of the 30S ribosomal subunit in prokaryotic cells, and it is transcribed as a single-stranded ribosomal RNA molecule. On the other hand, the 16SrDNA is the gene that encodes the 16SrRNA, and it consists of double-stranded chromosomal DNA. 16SrRNA sequencing is used to detect and identify bacterial pathogens in clinical specimens from patients with a suspicion of infection. 16SrDNA is applied to identify microorganisms and determine microbial communities.

Recently, molecular techniques, including 16SrRNA sequencing, have been used to reach fast and precise results in reference or research laboratories, and such methods are now recommended in challenging conditions such as PA infection.

Furthermore, our results showed that beta-lactam antibiotics were used in the majority of cases (91%) as a selective drug, with intravenous and oral penicillin being used in half of the cases, followed by amoxicillin, amoxicillin–clavulanic acid, and ampicillin. This result is predictable, as antibiotic resistance is not considered a problem in actinomycosis.

Usually, *Actinomyces* spp. are susceptible to beta-lactams, and in particular, penicillin G and amoxicillin are considered the desirable drugs for actinomycosis treatment. Since *Actinomyces* spp. do not produce beta-lactamases, combining amoxicillin with beta-lactam inhibitors such as clavulanic acid is not usually necessary unless there are co-pathogens such as Enterobacteriaceae presumed in the infection (3, 65, 66).

Furthermore, in a retrospective analysis from China, 46% of the cases were treated with penicillin G (60). A recent study was conducted in Turkey on 37 PA patients, and it was reported that most cases (73%) were treated with penicillin G and ampicillin-sulbactam, 13% with cefuroxime and ceftriaxone, and 5.4% with clarithromycin, levofloxacin, and moxifloxacin (1). Contrastingly, ampicillin/sulbactam was used only in one case. Cefuroxime, erythromycin, and clarithromycin were used in one case. Additionally, macrolides are considered useful alternatives (65).

Furthermore, the presence of *A. actinomycetemcomitans*, *Prevotella* spp., *P. acnes*, *F. nucleatum*, *Peptostreptococcus* spp., and *Fusobacterium* spp. alongside *Actinomyces* spp. was reported in some cases. It seems that treatment with beta-lactam agents can effectively lead to the successful treatment of PA. However, it is important to note that metronidazole has no *in vitro* activity against *Actinomyces* (65). Therefore, combination therapy with penicillin G was observed in one case of PA, where *P. acnes* and *F. nucleatum* were grown simultaneously. Hoca et al. reported the combination of metronidazole with other antibiotics in four cases of PA with co-infection (1).

Treatment with piperacillin–tazobactam was observed in only one case and could be related to the fact that although piperacillin–tazobactam, meropenem, and imipenem are considered active against *Actinomyces* spp., their use should be limited to prevent the acquisition of resistant flora, as they have broad-spectrum effects (65). Interestingly, feneticilline, which is not approved, was used in one case from the Netherlands after initial treatment with penicillin G. Finally, antibiotic therapy is administered for a prolonged duration because of the chance of recurrence in PA. Patients with no surgical intervention and a shorter period of 3 months of antibiotic therapy are at a higher risk of recurrence (13, 58, 67). Furthermore, no study currently suggests the period for follow-up of recurrent infection, although some studies suggested 3 months, 6 months, and 1-year follow-ups (9, 60, 67, 68). In summary, the treatment duration should be implemented in each case based on the main factors, such as severity and possible changes in the follow-up imaging.

### Limitations

In the current study, we only used available studies on PubMed/Medline, and only English studies were included. Therefore, the relevant publications decreased. Additionally, discussing the bias, risks, and individual limitations in the studies was not possible, as they were not reported.

## Conclusion

PA is a rare form of infection that is challenging to diagnose due to its non-specific symptoms, failure to detect pathogens, and resemblance to lung cancer. Although it can show similar imagining results as malignancy, which should be differentiated by the presence of nodules, in the sinus tract on the chest wall. Radiological techniques can be helpful in diagnosing PA but have their limitations. The limited available information about PA means that it can be easily confused with other diseases, leading to unnecessary surgeries.

Therefore, clinicians should be aware of the overlapping of signs and symptoms between PA and lung cancer. As antibiotic therapy may be adequate to treat this lung infection, biopsy specimen and histopathological examination should be considered before any surgical operation (i.e., lobectomy).

## Data availability statement

The raw data supporting the conclusions of this article will be made available by the authors, without undue reservation.

## Author contributions

AK: Writing – original draft, Writing – review & editing. NA: Writing – review & editing. SM: Writing – review & editing.
